# Effects of a 12 Week Ketogenic Diet Intervention on Obese and Overweight Females with Glucose and Lipid Metabolism Disturbance

**DOI:** 10.3390/nu16234218

**Published:** 2024-12-06

**Authors:** Grzegorz Klonek, Grzegorz Zydek, Robert Roczniok, Mariusz Panek, Adam Zając, Małgorzata Magdalena Michalczyk

**Affiliations:** 1 Institute of Sport Science, Academy of Physical Education in Katowice, Mikolowska 72a, 40-065 Katowice, Poland; klonek.grzegorz@gmail.com (G.K.); g.zydek@awf.katowice.pl (G.Z.); r.roczniok@awf.katowice.pl (R.R.); m.h.panek@gmail.com (M.P.); a.zajac@awf.katowice.pl (A.Z.); 2 Lenacor Sp. z o.o., Zagórska 73, 42-500 Bedzin, Poland

**Keywords:** ketogenic diet, females, hyperglycaemia, obese, overweight

## Abstract

Background/Objectives: We evaluated the effects of a 12-week hypocaloric ketogenic diet (KD) on glucose and lipid metabolism, as well as body mass, in overweight, obese, and healthy-weight females. One hundred adult females completed the study, including 64 obese (97.99 ± 11.48 kg), 23 overweight (75.50 ± 5.12 kg), and 11 with normal body mass (65.93 ± 3.40 kg). All participants followed a KD consisting of less than 30 g of carbohydrates, approximately 60 g of protein, and 140 g of fat per day (80% unsaturated and 20% saturated fat). Methods: Glucose (Gl), insulin (I), glycated haemoglobin (HBA1c), HOMA-IR, triglycerides (TG), and high-density lipoprotein cholesterol (HDL-C) were measured before and after the intervention. Additionally, body mass (BM), BMI (Body Mass Index), waist circumference (WC), hip circumference (HC), and thigh circumference (TC) were recorded. Results: After 12 weeks of the KD, significant improvements were observed in GL, I, TG, HDL-C, HOMA-_IR_ across all groups. Also BM, BMI, TC, WC, and HC were significantly reduced in all participants. Notably, obese participants showed greater reductions in all variables compared to overweight and healthy-weight females. Conclusions: A 12-week KD led to more pronounced improvements in biochemical markers and body mass in obese females compared to other groups. A KD may be particularly beneficial for obese females with hyperglycaemia, hyperinsulinemia, and lipid profile disturbances.

## 1. Introduction

Obesity and overweight are currently among the most significant global health issues [1,2]. Obesity is defined as a chronic disease with complex etiology, which is defined as abnormal or excessive accumulation of fat that poses a risk to health. The World Health Organization defines obesity as having a BMI of over 30 [1]. Being obese or overweight is contributes to numerous health issues [2]. High levels of body fat increase the risk of dyslipidaemia, Type 2 Diabetes (T2DM) 2 diabetes or insulin resistance, cardiovascular disease, and cancers such as colorectal, pancreatic, liver, and post-menopausal breast cancer [3,4,5,6]. The most common complications associated with obesity associated with cardiovascular system include hypertension, coronary artery disease, heart attacks, heart failure and stroke. Also liver and gastrointestinal problem such as Non-Alcoholic Fatty Liver Disease (NAFLD) [4] and reproductive and hormonal disorders such as infertility, polycystic ovary syndrome (PCOS) may appear in obese and overweight people [5]. Recent studies show a correlation between obesity and the development of sleep apnea, asthma and breathing disorders [7]. Therefore, effective treatment strategies for obesity and overweight are urgently needed [2,6,8].

For many years, researchers have agreed that the obesity and overweight epidemic is primarily caused by poor eating habits, particularly the overconsumption of carbohydrates, including sweets, fast foods, and sweetened drinks [8,9,10,11,12]. A high carbohydrate intake, combined with a sedentary lifestyle, promotes fat accumulation, leading to overweight and obesity [10,11,12]. Excess body fat is frequently accompanied by insulin resistance (IR), defined as impaired insulin-mediated glucose regulation [13]. In response, the body produces more insulin to maintain normal blood glucose levels [14]. IR is a precursor to Type 2 Diabetes (T2DM) 2 diabetes and is associated with other metabolic disorders. It is also linked to inflammation, hormonal imbalances, and cellular stress [3]. In obese individuals with IR, dyslipidaemia is common, with increased hepatic production of triglycerides and very-low-density lipoprotein (VLDL), and reduced HDL-C levels.

In recent years, the most widely recommended strategy for fat reduction by healthcare professionals has been a hypocaloric, low-fat, and low-carbohydrate ketogenic diet (KD) [14,15,16,17,18,19]. The KD is particularly effective for obese individuals with IR and dyslipidaemia [15,16,20]. The KD emphasizes high fat consumption (up to 70% of daily calorie intake) and restricts carbohydrate intake to 20–50 g per day, with moderate protein consumption [17,19,21]. These macronutrient shifts induce changes in digestion and metabolism, enhancing fat digestion and cellular processes like lipolysis and β-oxidation [13]. On a KD, fats are broken down into free fatty acids, which are then oxidized into ketone bodies (KBs) in the liver, providing energy, particularly for neurons and muscles.

Numerous studies have confirmed the KD’s positive impact on body composition and metabolic health [12,15,22,23]. Regardless of its duration, from 1 week to 12 months, the KD consistently results in weight loss and metabolic improvements [13,15,24]. Studies on overweight and obese individuals following the KD report fat loss, reduced glucose levels, and improved lipid profiles [10]. Recent findings indicate that the KD leads to greater reductions in body mass, fasting blood glucose, HBA1c1c, and TG, as well as an increase in HDL-C, compared to other diets such as low-fat or balanced diets [10,13,14].

Several randomized trials have demonstrated that low-carbohydrate, high-fat diets (LCHFD), particularly the KD, are more effective than low-fat, high-carbohydrate diets (LFHCD) in reducing triglycerides and LDL cholesterol, and increasing HDL-C cholesterol in individuals with abdominal obesity and dyslipidaemia [15,25]. Proposed mechanisms include reduced caloric intake, increased satiety from protein, hormonal changes regulating appetite, and the possible appetite-suppressing effects of KBs [19,26]. Interestingly, KB levels typically reach around 3 mmol/L during the KD, without changes in pH, confirming the diet’s safety [20,27,28]. In contrast, fasting can raise KB levels to 10 mmol/L, potentially leading to acidosis [20,26]. An increase in KB levels (from 0.3 to 1.5 mmol/L) after just a few days on the KD is linked to appetite suppression [28]. This appetite-suppressing effect, along with hormonal changes (e.g., ghrelin, leptin, neuropeptide Y, and PYY), contributes to fat loss [28]. Additionally, the KD increases resting energy expenditure (REE) and the rate of lipolysis [28].

In this study, we evaluated the effects of a 12-week KD on fasting glucose, insulin, glycated haemoglobin, HOMA-IR, HDL-C, and TG levels in 100 females, categorized as overweight, obese, or healthy-weight. We also assessed changes in body mass (BM), waist, hip, and thigh circumferences.

## 2. Material and Methods

### 2.1. Study Sample

**Figure 1:**
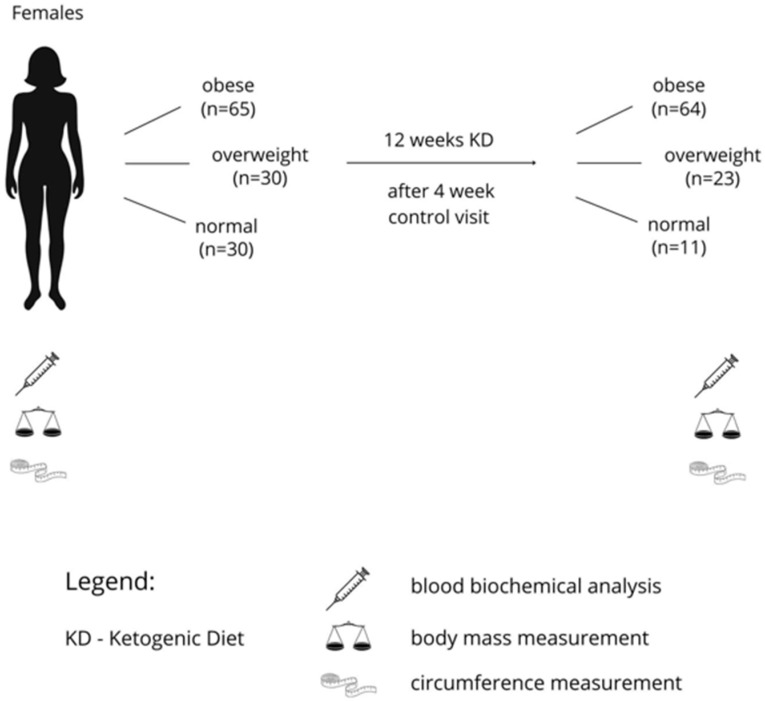
Diagram illustrating the course of the study.

The study was conducted in collaboration with the Nutrition Department at the Academy of Physical Education in Katowice and certified dietitians from the Lenacor Diet Clinic in Będzin, Poland. These institutions have had a longstanding partnership [10]. Participants were recruited from females who independently visited the clinic between January 2023 and December 2023. Initially, 125 adult females were enrolled in the study (n = 65 in the obese group, n = 30 in the overweight group, and n = 30 in the normal body mass group) (Figure 1). After 12 weeks, 63 obese (body mass 97.99 ± 11.48 kg, height 167.69 ± 5.26 m, age 41 ± 6 y), 23 overweight (body mass 75.50 ± 5.12 kg, height 163.74 ± 5.16 cm, age 41± 6 y) and 11 with normal body mass (body mass 65.93 ± 3.40 kg, height 167.54 ± 3.75 cm, age 39 ± 10 y) completed the study. The remaining participants did not attend the final laboratory session for blood tests and body weight measurements. The inclusion criteria were as follows: age between 20 and 50 years; before menopause, no engagement in any diet or food restriction in the past six months; and participation in mild exercise more than twice a week. Exclusion criteria included: menopause syndrome, pharmacological treatment for insulin resistance or dyslipidaemia; hypertension (systolic blood pressure > 140 mmHg and/or diastolic blood pressure > 90 mmHg, or use of antihypertensive medication); use of supplements known to affect lipid or glucose profiles; energy expenditure from physical activity > 1000 kcal/week; multiple allergies; celiac disease or other intestinal disorders; any condition that could limit mobility and make laboratory visits difficult; and life-threatening diseases or conditions that could affect adherence to the study. Before the experiment, all participants were informed about the study’s objectives, risks, and benefits, as well as their right to withdraw at any time. Each participant read and signed an informed consent form. The study protocol was approved by the local Ethics Committee at the Academy of Physical Education in Katowice, Poland (Ethics reference: KB-2/2021).

### 2.2. Dietary Procedures

The dietary intervention lasted 12 weeks. Each participant received ketogenic diet (KD) with mean ±1800 kcal daily caloric intake (Table 1) which was below their total daily energy expenditure (TDEE). The TDEE was calculated using the formula: TDEE = Activity Factor (AF) × Resting Metabolic Rate (RMR) [29]. RMR was calculated using Harris- Benedict’s formula. The proper body mass was calculated according to the Lorenz formula. The AF was determined based on indicators for adults with low physical activity (AF = 1.4) [30]. Before the experiment, all participants followed a typical Western-style diet [12,31] and had no previous experience with calorie-restricted or low-carbohydrate diets. The composition of each subject’s diet was analysed using DIETETYK 6.0 software (Jumar, Poland).

### 2.3. Diet Composition

The composition of the ketogenic diet (KD) is presented in Table 1. KD meal plans were developed for 24-h menus, covering all seven days of the week, with two main meals consumed between 6:00 a.m. and 8:00 M.P. The KD was based on high-quality food products [10]. Participants consumed healthy fats, primarily monounsaturated fatty acids from sources like olive oil, canola oil, dairy products, and nuts. The diet included both *n*-6 and *n*-3 polyunsaturated fatty acids in a ratio of 5-6:1. Fatty fish such as mackerel, salmon, herring, and sardines, rich in *n*-3 fatty acids, were incorporated alongside fatty meats and eggs. Protein intake was aligned with reference values for adults, amounting to approximately 1.5 g per kg of body mass [32]. The ketogenic ratio in the diet was 1:1.5. The meals mainly consisted of fish, beef, pork, veal, lamb, poultry, eggs, and cheese, along with olive oil, canola oil, butter, nuts, and raw green vegetables [10]. Hot beverages were limited to unsweetened tea, coffee, or herbal extracts, and participants were instructed to drink at least 2.5 litres of water per day. Artificial sweeteners such as saccharin, cyclamate, acesulfame, aspartame, and sucralose were prohibited. Additionally, participants consumed about 200 mL of beef or chicken broth daily to provide essential vitamins and minerals, particularly potassium and sodium. Foods such as sweetened milk, fruit yogurt, whole grain or white bread, pasta, white rice, sweets, instant tea or barley coffee, as well as any foods or drinks containing alcohol and sugar, were strictly prohibited.

### 2.4. Supplements

Participants received one tablet of a multivitamin-mineral supplement (Centrum, Pfizer, Wiedeń, Austria) daily, along with one tablet of vitamin D3 (2000 IU) and one tablet of calcium carbonate (1500 mg).

### 2.5. Diet Control

During the 12-week intervention, subjects followed daily menus and prepared their own meals. They were provided with a detailed list of permitted and prohibited foods on the ketogenic diet (KD) and consulted with dietitians during follow-up visits every two weeks. To enhance dietary adherence, participants measured their blood ketone levels weekly in the morning before meals using the Optimum Xido Neo device (Abbott, Oxon, UK).

### 2.6. Experimental Design

Fasting blood samples were taken from all participants before and after the 12-week study to assess biochemical variables. Additionally, anthropometric measurements were conducted to determine body composition variables.

### 2.7. Biochemical Analysis

Before and after the intervention, the following biochemical variables were measured: β-hydroxybutyrate (β-HGB, mmol/L), glucose (Gl, mmol/L), insulin (I, μg/dL), HBA1c1c (mg/L), triglycerides (TG, mg/dL), and high-density lipoprotein cholesterol (HDL-C, mg/dL). β-HGB measurements were performed using Randox UK diagnostic kits (Ranbut, Crumlin, County Antrim, UK), while Beckman Coulter diagnostic kits were used for the other variables (Gl—OSR6221, I—OSR33410, Variant II Turbo HBA1c1c Kit 2.0—270-2455EX; TG—OSR66118; HDL-C—OSR6687). HOMA-IR was calculated using the formula: (glucose concentration in mmol/L) × (insulin concentration in μg/dL) ÷ 22.5 [33].

### 2.8. Body Mass and Circumference Measurements

Before and after the experiment, participants’ body mass (BM), waist circumference (WC), hip circumference (HC), and thigh circumference (TC) were measured under controlled laboratory conditions. WC was measured standing, approximately 0.5 cm above the midpoint between the lowest rib and the iliac crest. HC was assessed at the level of the hip bones, while TC was measured 2 cm below the umbilicus [34]. Participants wore only underwear during these anthropometric measurements. BM was evaluated using multifrequency bioimpedance analysis (MF-BIA) with the InBody 220 device (Biospace Co., Ltd., Seoul, Republic of Korea).

### 2.9. Statistical Analysis

All analyses were performed using the Statistica 13.1 software package. The normality of distributions was assessed with the Shapiro-Wilk test, while Levene’s test was used to verify the homogeneity of variances, and Mauchly’s test was employed to check for sphericity. The results are presented as means with standard deviations, standard errors, and 95% confidence intervals. One-way analysis of variance, non-parametric Kruskal-Wallis, and multivariate repeated measures ANOVA were used to compare differences between the variables under consideration. Effect sizes for main effects and interactions were determined using partial eta squared (η^2^), with thresholds for small (0.01 to 0.059), moderate (0.06 to 0.137), and large (>0.137) effects. In cases of significant main effects or interactions, post-hoc comparisons were performed using Bonferroni’s test. Statistical significance for differences between load types and muscle sides was set at *p* < 0.05. Additionally, effect sizes (Cohen’s d) were calculated and interpreted as large (d > 0.8), moderate (d between 0.5 and 0.8), and small (d < 0.5).

## 3. Results

The study included 98 participants. Analyses began by calculating basic descriptive statistics and 95% confidence intervals (CI) for the variables analyzed by group. The results are presented in Table 2.

The results of the analysis of variance for the interaction term group × before-after were significant F = 12.77; *p* < 0.0001; η^2^ = 0.21. The interaction analysis indicated a statistically significant decrease in body mass across all groups (*p* < 0.001). An analysis of variance (ANOVA) for BMI revealed significant interaction effects between the group and pre- and post-measurements (F = 11.98; *p* = 0.00002; η^2^ = 0.20).The interaction analysis indicated a statistically significant decrease in body mass across all groups (*p* < 0.001) except for two interactions between BMI before for the normal group compared to BMI after for the overweight group ( *p* = 0.99), and BMI before for the overweight group compared to BMI after for the obesity group (*p* = 0.99). For HC, the analysis of variance showed no significant differences for the interaction group × before-after, F = 2.72; *p* = 0.07. Similarly, for WC, significant differences were observed for the interaction group × before-after, F = 7.29; *p* = 0.0011; η^2^ = 0.13. The interaction analysis indicated a statistically significant decrease in WC across all groups (*p* < 0.001). Initially, significant differences were observed between the normal body mass group and the overweight group (*p* = 0.0043; d = 1.50). For Gl, no significant differences were found for the interaction group × before-after, F = 2.79; *p* = 0.066. However, for I levels, significant differences were observed for the interaction group before-after, F = 5.33; *p* = 0.0064; η^2^ = 0.10. The interaction analysis indicated a statistically significant decrease in I levels across all groups (*p* < 0.001). Before the experiment, significant differences in I levels were observed between the normal and obese groups (*p* = 0.045; d = 0.69), and between the overweight and obese groups (*p* < 0.0001; d = 0.96). After the experiment, these differences were no longer significant (*p* = 0.57 for normal vs. obese; *p* = 0.99 for overweight vs. obese).The ANOVA for BMI demonstrated significant differences in the interaction between group and time (before and after measurements), yielding F = 11.98, *p* = 0.00002, and η^2^ = 0.20.The interaction analysis revealed a significant decrease in HOMA-IR levels only for the overweight group (*p* = 0.044; d = 1.33) and the obese group (*p* < 0.0001; d = 1.49). Before the experiment, significant differences in HOMA-IR levels were found between the normal and obese groups (*p* = 0.0018; d = 0.88) and between the overweight and obese groups (*p* < 0.0001). After the experiment, these differences were no longer significant (*p* = 0.99 for normal vs. obese; *p* = 0.99 for overweight vs. obese). For HBA1c levels, significant differences were observed for the interaction group × before-after, F = 4.64; *p* = 0.012; η^2^ = 0.089. A statistically significant decrease in HBA1c levels was observed only in the overweight group (*p* = 0.0073; d = 0.55) and the obese group (*p* < 0.0001; d = 1.29). Before the experiment, significant differences in HBA1c levels were noted between the normal and obese groups (*p* = 0.0002; d = 1.40) and between the overweight and obese groups (*p* < 0.0001; d = 0.59). After the experiment, these differences were no longer significant (*p* = 0.86 for normal vs. obese; *p* = 0.99 for overweight vs. obese). An analysis of HDL-C levels indicated significant interaction effects between the group and time (before and after measurements), with F = 4.57, *p* = 0.013, and η^2^ = 0.088. A statistically significant increase in HDL-C levels was observed in the overweight (*p* < 0.0001; d = 1.01) and obese groups (*p* < 0.0001; d = 0.94). Before the experiment, significant differences in HDL-C levels were observed between the normal and obese groups (*p* = 0.0001; d = 1.85) and between the overweight and obese groups (*p* < 0.0001; d = 1.70). TG levels were analyzed using ANOVA, which revealed significant interaction effects between the group and time (before and after measurements), with F = 11.54, *p* = 0.00003, and η^2^ = 0.20. A statistically significant decrease in TG levels was noted only in the overweight group (*p* < 0.0001; d = 0.90) and the obese group (*p* < 0.0001; d = 1.10). Before the experiment, significant differences in TG levels were observed between the normal and obese groups (*p* = 0.0001; d = 1.21) and between the overweight and obese groups (*p* = 0.0001; d = 1.35). After the experiment, these differences were no longer significant (*p* = 0.99 for both comparisons). For BHB levels, significant differences were found for the interaction group × before-after, F = 3.63; *p* = 0.030; η^2^ = 0.071. A statistically significant increase in BHB levels was observed across all analyzed groups (*p* < 0.0001). Before the experiment, significant differences in BHB levels were observed between the normal and obese groups (*p* = 0.0001; d = 0.19) and between the overweight and obese groups (*p* = 0.0001; d = 0.26). After the experiment, these differences were no longer significant (*p* = 0.99 for both comparisons).In subsequent analyses, the significance of the differences between the deltas (the difference in the results after minus before for the analyzed variables due to the group) was verified. For this purpose, one-way analysis of variance and Bonferroni’s multiple comparison tests were used, or, in the absence of normality of the distributions, Kruskal-Wallis nonparametric analysis of variance and multiple comparison tests were applied.

The results of the analysis of variance for the ΔBM mass results revealed significant differences. It was found that there was a statistically significant higher body mass reduction (experimental effect) in the obesity group than in the normal group (*p* = 0.0006; d = 1.14) and in the overweight group (*p* = 0.0005; d = 0.93). The results of the analysis of variance for the ΔBMI showed significant differences. It was found that there was a statistically significant higher BMI reduction (experimental effect) in the obesity group than in the normal group (*p* = 0.0001; d = 1.09) and in the overweight group (*p* = 0.0006; d = 0.91). An analysis of the variable ΔWC revealed that the obesity group experienced a statistically significantly greater reduction in waist circumference than the normal group (*p* = 0.0071; d = 1.21). The results for the ΔI after-before showed a statistically significant higher decrease in I levels in the obesity group than in the overweight group (*p* = 0.005; d = 0.77). The results for the ΔHOMA-IR showed a statistically significant higher decrease in ΔHOMA levels in the obesity group than in the overweight group (*p* = 0.004; d = 0.80). Results for the ΔHBA1c showed a statistically significant higher decrease in HBA1c levels in the obesity group than in the normal group (*p* = 0.020; d = 0.88). Results for the variable ΔHDL-C revealed that the overweight group had a statistically significant higher increase in HDL-C levels than the normal group (*p* = 0.041; d = 0.89). Results for the ΔTG found significant differences between the overweight group (*p* = 0.0001; d = 1.38) and the obesity group; there was a statistically significant higher decrease in TG levels than in the normal group, which showed an increase in TG levels (*p* < 0.0001; d = 1.78). Results for the variable ΔBHB indicated significant differences, but multiple comparison tests did not confirm the results from ANOVA (*p* > 0.05).

## 4. Discussion

In the present study, we observed the effects of 12 week ketogenic diet (KD) intervention on glucose and lipid metabolism, body mass, and body circumferences in female participants. The study included 64 obese, 23 overweight, and 11 women with normal body mass (Figure 1). From the start of the KD, multivitamin supplementation was introduced to prevent vitamin and mineral deficiencies, maintain a healthy electrolyte balance, and support the immune and antioxidant defence system [35]. We also ensured proper hydration, providing participants from all groups with adequate water intake. The results showed that after 12 weeks, the KD significantly reduced body mass and circumferences in all participants. These changes were accompanied by improvements in all measured biochemical variables, such as fasting glucose, insulin, HBA1c1c, triglycerides (TG), and HDL-C concentrations, particularly in the obese and overweight groups. The participants did not report any complications after 3 months of the diet. After the project ended, the participants used a low-carb diet and after a few weeks individually, they either stayed on the low-carb diet or switched to a mixed diet.

For decades, scientists worldwide have sought the most effective means to reduce obesity and overweight [10]. Many strategies have been proposed, including drug therapies, bariatric surgery, physical activity, and various dietary interventions [23]. Numerous clinical nutrition studies have focused on evaluating the physiological effects of different diets for treating obesity and overweight, including low-calorie diets, very-low-calorie diets, low-fat diets, low-carbohydrate diets, and the ketogenic diet (KD) [10,12,36,37,38,39]. Despite the proven effectiveness of the KD for weight loss in many studies, no clear guidelines have been established regarding the normal duration of the diet or a precise definition of acceptable foods during the KD. Studies have explored the effects of the KD on body mass reduction over periods ranging from a few days to several months [13,23]. These studies have used both the classic KD model and variations such as the Mediterranean and Spanish KDs [13,23].

Regardless of the duration or specific type of KD, studies consistently report significant weight loss. We observed similar results, particularly in obese participants, who significantly (*p* < 0.001) reduced their body mass from 97.99 ± 11.48 kg to 81.94 ± 10.95 kg. Overweight participants and those with normal body mass also showed significant (*p* < 0.001) reductions, from 75.50 ± 5.12 kg to 65.17 ± 4.36 kg and from 65.93 ± 3.40 kg to 57.42 ± 5.71 kg, respectively. These findings align with our previous study, where a 12-week low-calorie KD also resulted in significant body mass reductions in obese women, from 89.08 ± 14.68 kg to 75.36 ± 13.47 kg. Similarly, Shai et al. [40] reported significant weight loss after a two-year low-carbohydrate intervention, suggesting that this dietary approach could be recommended for patients with body mass disturbances. Similar results have been achieved by other researchers [36,41]. The metabolic consequences of weight loss include improved tissue sensitivity to insulin, reduced fasting glucose levels, and a reduced HOMA-IR index [42]. Dyslipidemia is also regulated [43].

The most likely mechanisms behind the body mass reduction observed during the KD include decreased insulin secretion by the pancreas and increased synthesis of ketone bodies (KBs), such as β-hydroxybutyrate, acetoacetate, and acetone, by the liver. Insulin levels in the blood are largely dependent on carbohydrate intake. When carbohydrate consumption is drastically reduced, as in the KD, insulin synthesis decreases, inhibiting fat storage in adipose tissue while increasing lipolysis. The higher concentration of KBs during the KD, typically rising from <0.5 mmol/L to about 2–3 mmol/L, further enhances lipolysis. At this point, it should be noted that the mechanism of appetite regulation during a ketogenic diet (KD) is a topic of ongoing discussion [26,44,45]. As previously mentioned, elevated ketone body (KB) levels suppress appetite and influence the secretion of appetite-regulating hormones, such as ghrelin and leptin [13,23,42]. At this point, it should be noted that the mechanism of appetite regulation during a ketogenic diet (KD) is a topic of ongoing discussion [26,44,45]. As previously mentioned, elevated ketone body (KB) levels suppress appetite and influence the secretion of appetite-regulating hormones, such as ghrelin and leptin. The KD affects both orexigenic and anorexigenic signals [44]. This is partly due to an increase in β-hydroxybutyrate (βHB) concentration, which elevates circulating adiponectin levels, γ-aminobutyric acid (GABA) in the brain, and phosphorylation of AMP-activated protein kinase—mechanisms that collectively have an anorexigenic effect. Conversely, by reducing the production of reactive oxygen species in the brain, the KD has an orexigenic effect.Additionally, βHB reduces the expression of neuropeptide Y, which plays a role in regulating food intake, increases postprandial cholecystokinin (CCK) secretion, and decreases ghrelin secretion [26,44]. It is also hypothesized that the appetite-suppressing effect of the KD may result from a reduction in butyrate production, which enhances glucagon-like peptide-1 (GLP-1) secretion. Notably, GLP-1 can also have an appetite-activating effect. Gut peptides such as ghrelin, peptide YY (PYY), GLP-1, and CCK regulate appetite throughout the day, with their secretion varying between meals [26]. In the initial days of the KD, there is an increase in βHB levels accompanied by changes in appetite-regulating hormones, such as ghrelin. Subjective feelings of hunger and the desire to eat initially increase during this phase [26,45]. After 2–3 weeks of ketosis, appetite-related sensations continue to rise, with both basal and postprandial ghrelin concentrations increasing. At the same time, basal GLP-1 and CCK plasma levels, as well as postprandial PYY and CCK levels, decrease. However, after 8 weeks on the KD, the increase in ghrelin secretion is suppressed [45], and by 12 weeks, there are no significant changes in plasma ghrelin concentrations [45].

During the KD, excessive KB production also leads to metabolic ketosis, a natural state for cells [8]. During the KD, β-hydroxybutyrate and acetoacetate serve as alternative energy sources, primarily for the brain. It’s worth noting that small amounts of KBs are produced even when the diet includes more than 50 g of carbohydrates, and these are quickly utilized by skeletal and cardiac muscles.

Recent research has revealed the pleiotropic effects of KBs, including their influence on gene expression in skeletal muscles and their role in reducing inflammation [46,47]. It is also believed that β-hydroxybutyrate, the most frequently synthesized ketone by the liver, directly scavenges hydroxyl radicals (∙OH), while indirectly improving mitochondrial efficiency through increased redox potential [48,49]. In this study, after 12 weeks of the KD (Table 3), we observed β-hydroxybutyrate concentrations of up to 1.5 mmol/L in participants, confirming that they were in ketosis [10].

In addition to strong scientific evidence for body mass reduction, equally robust evidence supports the use of the KD in regulating glucose metabolism disturbances, such as hyperglycaemia and hyperinsulinemia [29]. These findings have been demonstrated in numerous studies [13,23,35,36,38]. In our study, we also observed significant reductions in glucose, insulin, HBA1c concentrations, and HOMA-IR levels [10,13,23,40]. Among obese females, we recorded a significant reduction in GL (from 5.51 ± 0.54 mmol/L to 5.08 ± 0.56 mmol/L) and insulin levels (*p* < 0.001) from 17.30 ± 8.17 µlU/mL to 8.15 ± 2.98 µlU/mL, as well as HBA1c (*p* < 0.001) from 5.64 ± 0.37% to 5.18 ± 0.34%, and HOMA-IR (*p* < 0.001) from 4.32 ± 2.23 to 1.85 ± 0.73. In the overweight group, while GL levels showed a reduction, the change was not statistically significant (Table 2). However, insulin levels were significantly reduced (*p* < 0.001) from 10.13 ± 4.84 µlU/mL to 5.71 ± 1.80 µlU/mL, HBA1c (*p* < 0.001) from 5.28 ± 0.25% to 5.02 ± 0.39%, and HOMA-IR (*p* < 0.001) from 2.35 ± 1.24 to 1.28 ± 0.42. In females with normal body mass, only insulin levels were significantly reduced (*p* < 0.001) from 11.89 ± 5.89 µlU/mL to 4.95 ± 1.97 µlU/mL, while other variables, such as GL, HBA1cc, and HOMA-IR, were reduced but not significantly (Table 3). It is important to note that at the start of the study, these biochemical variables were already within the normal range for this group. Our results are consistent with those reported by other authors. In a previous study, after 12 weeks of a low-calorie ketogenic diet, we also observed significant reductions in insulin (from 14.12 ± 4.75 µlU/mL to 6.61 ± 2.63 µlU/mL), GL (from 5.94 ± 0.56 to 4.74 ± 0.9 mmol/L), HBA1c (from 5.87 ± 0.94 to 5.38 ± 0.74 mg/L), and HOMA-IR (3.73 ± 1.2 vs. 1.38 ± 0.63) in obese females [10]. Similarly, Shai et al. [40], after 24 months of a Mediterranean low-carbohydrate diet intervention, recorded significant reductions in fasting plasma GL and insulin levels, as well as reductions in HBA1c and HOMA-IR. The authors suggest that these dietary strategies should be considered in clinical practice, tailored to individual preferences and metabolic needs [40]. Paoli et al. [23] also demonstrated that after 12 weeks of the KD, participants experienced reductions in fasting blood glucose levels, from approximately 93 mg/dL to 85 mg/dL. Likewise, Peres-Guisado et al. [41], after 12 weeks of the “Spanish Ketogenic Mediterranean Diet” (SKMD), observed a decrease in fasting glucose levels from 118.57 mg/dL to 90.14 mg/dL in patients with the metabolic syndrome.

The efficacy of the KD in reducing glucose and insulin concentrations arises from its drastic reduction of carbohydrates. Lowering daily carbohydrate intake alters cellular metabolism, primarily by inhibiting pancreatic insulin secretion, which regulates blood glucose levels. Insulin receptors are predominantly located in adipose and muscle tissues, where insulin binding activates glucose transporters like GLUT4 [50]. These transporters then fuse with the cell membrane and facilitate the movement of glucose from the bloodstream into fat or muscle cells, promoting lipogenesis. In summary, carbohydrate intake directly affects both blood glucose and insulin levels and adipose tissue synthesis [51,52].

One of the most significant positive effects of the KD, confirmed in many studies, is the regulation of lipid profile disturbances. Numerous authors have observed that after a few weeks of KD, HDL cholesterol levels increase while triglyceride (TG) levels decrease [10,23,40,41]. For example, Peres-Guisado et al. [41] reported that after 12 weeks of the SKMD in participants with metabolic syndrome, HDL levels increased (from 42.81 mg/dL to 58.71 mg/dL), while TG levels decreased (from 232.64 mg/dL to 111.21 mg/dL). In our previous study [10], after 12 weeks of a low-calorie ketogenic diet, we also observed significant reductions in TG (213.45 ± 63.60 mg/dL vs. 129.13 ± 46.23 mg/dL) and increases in HDL-C levels (36.71 ± 4.42 mg/dL vs. 52.99 ± 7.77 mg/dL) in obese females. In the present study, we observed similar changes (Table 3). In both obese and overweight females, TG levels were significantly reduced (*p* < 0.001), from 140.28 ± 55.06 mg/dL to 92.52 ± 27.31 mg/dL in the obese group, and from 151.30 ± 79.70 mg/dL to 97.74 ± 28.30 mg/dL in the overweight group. Simultaneously, HDL-C levels significantly increased (*p* < 0.001), from 52.61 ± 10.99 mg/dL to 59.92 ± 11.32 mg/dL in the obese group and from 52.09 ± 10.98 mg/dL to 62.97 ± 10.76 mg/dL in the overweight group.

The diet consumed by our participants was rich in omega-3 and omega-9 fatty acids, likely contributing to these changes [10]. According to Peres-Guisado et al. [41], the high content of olive oil and fish in the KD, which are sources of omega-9 and omega-3 fatty acids, may influence changes in lipid profiles. Specifically, the omega-3 fractions EPA and DHA, found in fish, meat, and eggs, are known to reduce TG concentrations in blood plasma by inhibiting their resynthesis in the liver and enterocytes. EPA and DHA modulate at least four transcription factors involved in hepatic TG and very low-density lipoprotein (VLDL) synthesis, particularly by inhibiting sterol regulatory element-binding protein (SREBP-1) and liver X receptors (LXR) and activating farnesol X receptor (FXR), thereby limiting the expression of enzymes responsible for lipogenesis [53,54].

The last aspect we measured in our experiment was the change in waist, hip, and thigh circumferences. A significant decrease in hip and thigh circumferences was observed in all participants (Table 3), clearly indicating a reduction in fat tissue in these areas. Our results are consistent with those presented by other authors [55,56]. Peres-Guisado et al. [41], who used the Spanish Ketogenic Mediterranean Diet in patients with metabolic syndrome, also recorded waist circumference changes from 114.01 cm to 98.59 cm. In our previous study, we achieved similar results [10]. After 12 weeks of a low-caloric ketogenic diet, we observed decreases in WC (101.04 ± 11.86 cm vs. 87.34 ± 9.50 cm), HC (112.82 ± 9.89 cm vs. 101.21 ± 7.42 cm), and TC (64.57 ± 6.36 cm vs. 56.91 ± 6.36 cm) in obese females [10]. The reduction of fat in the hip and thigh areas may have a positive impact on hormonal imbalances related to oestrogen, progesterone, and growth hormone levels [34,57].

### Strengths and Limitations

The our studies are primarily long-term, which represents a significant advantage of our research. Additionally, the large sample size enhances the reliability of the results, providing further evidence for the effectiveness of using a ketogenic diet (KD) in reducing body weight and metabolic disorders. A third important aspect of our research is that it was conducted on a group of women, whereas most previous studies have focused on men. However, a limitation of our study is that we did not monitor the participants’ menstrual cycle through hormonal markers, and we lack a control group consuming a typical Western diet. Additionally the lack of body fat measurement and reliance solely on circumference measurements to estimate body fat reduction is also a limitation of our study.

## 5. Conclusions

In conclusion, our findings support the effectiveness of a 12-week KD in inducing significant reductions in body mass and enhancing metabolic health in females. These results suggest that a ketogenic approach may be beneficial for managing obesity and metabolic disturbances associated with overweight. Future studies should explore long-term effects of KD on general health and exercise tolerance.

## Figures and Tables

**Table 1:** Average macronutrients and total energy content of the ketogenic diet (KD) reported during the study.

Contents	KD- Normal Group ±Mean	KD- Overweight Group ±Mean	KD- Obese Group ±Mean
RMR, kcal TDEE	±1350 ±1890	±1320 ±1850	±1340 ±1880
TEI, kcal	1800	1800	1800
Carbohydrate, %	8	8	8
Carbohydrate, g	±35	±35	±35
Fiber, g	±15	±15	±15
Proteins, %	20	20	20
Proteins, g	±90	±90	±90
Proteins, g/kg bm/d	±1.5	±1.6	±1.5
Fat, %	72	72	72
Fat, g	±145	±145	±145
SFA, g	±30	±30	±30
MUFA, g	±93	±93	±93
PUFA, g	±23	±23	±23
*n*-6, g	±19	±19	±19
*n*-3, g	±4	±4	±4
EPA and DHA, g	±3	±3	±3
*n*-6/*n*-3	5:1	5:1	5:1
Cholesterol, g	±500	±500	±500

Note: RMR—Resting Metabolic Rate, TDEE—Total Daily Energy Expenditure, TEI—Total Energy Intake, SFA—Saturated Fatty Acids, MUFA—Monounsaturated Fatty Acids, PUFA—Polyunsaturated Fatty Acids, EPA—Eicosapentaenoic Acids, DHA—Docosahexaenoic Acids, *n*-3—Omega 3, *n*-6—Omega 6, kg bm/d—kg of body mass/day.

**Table 2:** Basic descriptive statistics for the analyzed variables.

Variables	Group					
	Normal N = 11 BMI m ± SD (95%)CI		Overweight N = 23 BMI m ± SD (95%)CI		Obesity N = 64 BMI m ± SD (95%)CI	
	Before	After	Before	After	Before	After
	m ± SD (95%)CI	m ± SD (95%)CI	m ± SD (95%)CI	m ± SD (95%)CI	m ± SD (95%)CI	m ± SD (95%)CI
Body mass [kg]	65.93 ± 3.40 *** (63.64:68.21)	57.42 ± 5.71 *** (53.58:61.26)	75.50 ± 5.12 ^###^ (73.29:77.72)	65.17 ± 4.36 ^###^ (63.29:67.06)	97.99 ± 11.48 ^^^ (95.12:100.85)	81.94 ± 10.95 ^^^ (79.20:84.67)
BMI	23.48 ± 0.88 *** (22.89:24.08)	20.40 ± 1.20 *** (19.60:21.21)	28.00 ± 1.19 ^###^ (27.49:28.51)	24.32 ± 1.45 ^###^ (23.69:24.95)	34.89 ± 4.18 ^^^ (33.84:35.93)	29.15 ± 3.78 ^^^ (28.21:30.10)
HC [cm]	98.55 ± 4.13 (95.77:101.32)	90.45 ± 4.91 (87.16:93.75)	107.78 ± 5.65 (105.34:110.23)	97.39 ± 4.12 (95.61:99.17)	115.55 ± 9.63 (113.14:117.95)	103.13 ± 6.30 (101.55:104.70)
WC [cm]	83.82 ± 7.47 *** (78.80:88.83)	75.64 ± 4.13 *** (72.86:78.41)	94.43 ± 7.01 ^###^ (91.40:97.47)	81.78 ± 5.54 ^###^ (79.39:84.18)	105.50 ± 9.11 ^^^ (103.22:107.78)	90.08 ± 7.70 ^^^ (88.16:92.00)
TC [cm]	58.45 ± 2.77 (56.59:60.32)	51.18 ± 3.16 (49.06:53.30)	59.63 ± 6.64 (56.76:62.50)	53.00 ± 5.12 (50.79:55.21)	63.52 ± 8.08 (61.49:65.56)	56.60 ± 7.27 (54.77:58.43)
Hb [g/dL]	13.52 ± 0.18 (13.39:13.64)	13.33 ± 0.48 (13.00:13.65)	13.83 ± 0.81 (13.48:14.19)	13.63 ± 0.65 (13.34:13.91)	13.44 ± 0.85 ^^^ (13.23:13.65)	13.86 ± 0.71 ^^^ (13.68:14.04)
GL [mg/dL]	86.72 ± 12.99 (77.99:95.44)	83.45 ± 8.79 (77.55:89.36)	92.13 ± 10.35 (87.65:96.61)	90.52 ± 7.01 (87.49:93.55)	99.23 ± 9.66 (96.82:101.65)	91.37 ± 10.01 (88.87:93.87)
I [μIU/mL]	11.89 ± 5.89 *** (7.93:15.85)	4.95 ± 1.97 *** (3.62:6.27)	10.13 ± 4.84 ^###^ (8.04:12.22)	5.71 ± 1.80 ^###^ (4.93:6.49)	17.30 ± 8.17 ^^^ (15.26:19.34)	8.15 ± 2.98 ^^^ (7.40:8.89)
GL [mmol/L]	4.82 ± 0.72 (4.33:5.30)	4.64 ± 0.49 (4.31:4.96)	5.12 ± 0.57 (4.87:5.37)	5.03 ± 0.39 (4.86:5.20)	5.51 ± 0.54 ^^^ (5.38:5.65)	5.08 ± 0.56 ^^^ (4.94:5.21)
HOMA-_IR_	2.46 ± 1.04 (1.76:3.16)	1.01 ± 0.41 (0.74:1.29)	2.35 ± 1.24 ^#^ (1.81:2.89)	1.28 ± 0.42 ^#^ (1.10:1.46)	4.32 ± 2.23 ^^^ (3.76:4.87)	1.85 ± 0.73 ^^^ (1.67:2.04)
HbA1c [mmol/mol]	5.14 ± 0.25 (4.97:5.31)	4.94 ± 0.26 (4.77:5.11)	5.28 ± 0.25 ^##^ (5.17:5.39)	5.02 ± 0.39 ^##^ (4.85:5.19)	5.64 ± 0.37 ^^^ (5.55:5.73)	5.18 ± 0.34 ^^^ (5.09:5.26)
HDL-C [mg/dL]	70.91 ± 7.89 (65.61:76.21)	70.55 ± 7.38 (65.59:75.50)	52.09 ± 10.98 ^###^ (47.34:56.84)	62.97 ± 10.76 ^###^ (58.32:67.62)	52.61 ± 10.99 ^^^ (49.87:55.36)	59.92 ± 11.32 ^^^ (57.09:62.75)
TG [mg/dL]	70.91 ± 7.89 (65.61:76.21)	100.27 ± 8.87 (94.32:106.23)	151.30 ± 79.70 ^###^ (116.84:185.77)	97.74 ± 28.30 ^###^ (85.50:109.98)	140.28 ± 55.06 ^^^ (126.53:154.04)	92.52 ± 27.31 ^^^ (85.69:99.34)
BHB [mmol/dL]	0.53 ± 0.30 *** (0.32:0.73)	3.20 ± 0.75 *** (2.70:3.70)	0.48 ± 0.25 ^###^ (0.37:0.59)	2.54 ± 0.52 ^###^ (2.31:2.76)	0.62 ± 0.35 ^^^ (0.53:0.71)	3.10 ± 0.68 ^^^ (2.93:3.27)

*p* < 0.05 #; *p* < 0.01 ##; *p* < 0.001 ***, ^^^, ### Note: BMI—Body Mass Index, HC—hip circumference, WC—waist circumference, TC—thigh circumference, Hb—hemoglobin, GL—glucose, I—insulin, HDL-C—high density cholesterol, TG—triglicerydes, BHB—B-hydroxybutyrate.

**Table 3:** Basic descriptive statistics and 95% CI for the analyzed Δ effects.

Variables	Group			Results for Anova
	Normal N = 11 BMI m ± SD (95%)CI (min; max) 23.48 ± 0.88 (22.89:24.08) (21.33:24.39)	Overweight N = 23 BMI m ± SD (95%)CI (min; max) 28.00 ± 1.19 (27.49:28.51) (25.75:29.79)	Obesity N = 65 BMI m ± SD (95%)CI (min; max) 34.89 ± 4.18 (33.84:35.93) (30.09:47.45)	
	M ± SD (95% CI)	M ± SD (95% CI)	M ± SD (95% CI)	
ΔBody mass [kg]	−8.51 ± 4.21 ^###^ (−11.34 to −5.68)	−10.33 ± 2.77 *** (−11.53 to −9.13)	−16.05 ± 6.94 ***^,###^ (−17.78 to −14.32)	F = 12.77; *p* = 0.00001; ɳ2 = 0.21
ΔBMI	−3.08 ± 1.59 ^###^ (−4.14:−2.01)	−3.68 ± 1.04 *** (−4.13:−3.22)	−5.73 ± 2.54 ***^,###^ (−6.37:−5.09)	F = 1.99; *p* = 0.00002; ɳ2 = 0.20
ΔHC [cm]	−8.09 ± 5.70 (−11.92 to −4.26)	−10.39 ± 3.99 (−12.11 to −8.67)	−12.42 ± 6.90 (−14.15 to −10.70)	H (2, N = 98) = 3.84; *p* = 0.15
ΔWC [cm]	−8.18 ± 6.46 ^##^ (−12.52 to −3.84)	−12.65 ± 6.51 (−15.47 to −9.84)	−15.42 ± 5.89 ^##^ (−16.89 to −13.95)	H (2, N = 98) = 12.92; *p* = 0.0016
ΔTC [cm]	−7.27 ± 1.62 (−8.36 to −6.19)	−6.63 ± 3.81 (−8.28 to −4.98)	−7.97 ± 8.99 (−10.21 to −5.72)	H (2, N = 98) = 0.63; *p* = 0.72
ΔHb [g/dl]	−0.19 ± 0.49 ^#^ (−0.52 to 0.14)	−0.21 ± 0.67 ** (−0.50 to 0.08)	0.42 ± 0.87 **^,#^ (0.20 to 0.64)	H (2, N = 98) = 12.56; *p* = 0.0019
ΔGL [mg/dl]	−3.26 ± 9.44 (−9.61 to 3.08)	−1.61 ± 11.38 (−6.53 to 3.31)	−7.87 ± 11.95 (−10.85 to −4.88)	F = 2.79; *p* = 0.067
ΔI [μIU/mL]	−6.95 ± 5.22 (−10.45 to −3.44)	−4.42 ± 3.53 ** (−5.95 to −2.89)	−9.15 ± 6.80 ** (−10.85 to −7.46)	H (2, N = 98) = 9.95; *p* = 0.0069
ΔGL [mmol/L]	−0.18 ± 0.52 (−0.53 to 0.17)	−0.09 ± 0.63 (−0.36 to 0.18)	−0.44 ± 0.66 (−0.60 to −0.27)	F = 2.79; *p* = 0.067
ΔHOMA-_IR_	−1.44 ± 0.92 (−2.06 to −0.83)	−1.07 ± 0.95 ** (−1.48 to −0.66)	−2.46 ± 1.95 ** (−2.95 to −1.98)	H (2, N = 98) = 10. 91; *p* = 0.0043
ΔHbA1c [mmol/mol]	−0.19 ± 0.13 ^#^ (−0.28 to −0.10)	−0.27 ± 0.43 (−0.45 to −0.08)	−0.47 ± 0.34 ^#^ (−0.55 to −0.38)	H (2, N = 98) = 8.93; *p* = 0.012
ΔHDL-C [mg/dL]	−0.36 ± 11.32 ^ (−7.97 to 7.24)	10.88 ± 13.27 ^ (5.15 to 16.62)	7.31 ± 8.57 (5.17 to 9.45)	H (2, N = 98) = 6. 21; *p* = 0.045
ΔTG [mg/dL]	29.36 ± 13.85 ***^,###^ (20.06 to 38.67)	−53.57 ± 71.76 ***^,^^^^ (−84.60 to −22.53)	−47.77 ± 46.42 ^###,^^^^ (−59.36 to −36.17)	H (2, N = 98) = 24.10; *p* < 0.0001
ΔBHB [mmol/dL]	2.67 ± 0.75 (2.19 to 3.15)	2.06 ± 0.58 (1.81 to 2.31)	2.48 ± 0.75 (2.29 to 2.66)	H (2, N = 98) = 6.61; *p* = 0.038

*p* < 0.05 ^,#; *p* < 0.01 **,##; *p* < 0.001 ***, ^^^, ### Note: HC—hip circumference, WC—waist circumference, TC—thigh circumference, Hb—hemoglobin, GL—glucose, I—insulin, HDL-C—high density cholesterol, TG—triglicerydes, BHB—B-hydroxybutyrate.

## Data Availability

The original contributions presented in this study are included in the article. Further inquiries can be directed to the corresponding author.
